# Role of Phospholipases A_2_ in Vascular Relaxation and Sympatholytic Effects of Five Australian Brown Snake, *Pseudonaja* spp., Venoms in Rat Isolated Tissues

**DOI:** 10.3389/fphar.2021.754304

**Published:** 2021-10-21

**Authors:** Nhi Thuc Vuong, Timothy N. W. Jackson, Christine E. Wright

**Affiliations:** ^1^ Cardiovascular Therapeutics Unit, Department of Biochemistry and Pharmacology, University of Melbourne, Parkville, VIC, Australia; ^2^ Australian Venom Research Unit, Department of Biochemistry and Pharmacology, University of Melbourne, Parkville, VIC, Australia

**Keywords:** Australian brown snake venoms, brown snake paradox, *Pseudonaja* brown snake, phospholipase A2, sympatholyic effects, vascular relaxation, cardiac effects, varespladib

## Abstract

Human envenoming by Australian brown snakes (*Pseudonaja* spp.) may result in potentially life-threatening hypotension and subsequent cardiovascular collapse. There have been relatively few studies of the cardiovascular and sympathetic effects of *Pseudonaja* spp. venoms. In this study, we have examined the effects of venom from five brown snake species—*P. affinis*, *aspidorhyncha, inframacula, nuchalis,* and *textilis*—on cardiac inotropic and chronotropic responses, vascular tone, and sympathetic nerve-induced vascular contractions in rat isolated tissues. The role of phospholipases A_2_ (PLA_2_s) in venom-induced effects was assessed with the sPLA_2_ inhibitor varespladib. In rat isolated left and right atria, there were no physiologically relevant effects of *Pseudonaja* venoms (0.1–30 µg/ml) on left atrial force of contraction (inotropy) or right atrial rate (chronotropy). In contrast, in isolated small mesenteric arteries precontracted with a thromboxane mimetic, each of the five brown snake venoms (at 30 µg/ml) caused marked vasorelaxation (−60 to –90% of contractile tone). Pretreatment with varespladib (1 µM) significantly inhibited the vasorelaxation caused by *P. aspidorhyncha*, *P. nuchalis,* and *P. textilis* venoms. Electrically induced sympathetic nerve-mediated contractions of mesenteric arteries were significantly attenuated by only *P. textilis*, and *P. affinis* venoms (30 µg/ml) and these sympatholytic effects were inhibited by varespladib (1 µM). Based on their inhibition with the sPLA_2_ inhibitor varespladib, we conclude that PLA_2_ toxins in *P. aspidorhyncha*, *P. nuchalis*, and *P. textilis* venoms are involved in brown snake venom-induced vasorelaxation and the sympatholytic effects of *P. affinis*, and *P. textilis* venoms. Our study supports the promising potential role of varespladib as an initial (pre-referral) and/or adjunct (in combination with antivenom) therapeutic agent for brown snake envenoming.

## 1 Introduction

Venom is a functional trait, used by venomous species of snake to subdue prey animals or deter potential predators (Jackson and Fry, 2016). The venom itself is often described as a “cocktail” (Casewell et al., 2013) because it contains numerous toxin species, each of which may interact with a different component of the target organism’s physiology. Venom achieves its goal of subjugation or deterrence by virtue of these toxins interfering with the normal functioning of various regulatory (homeostatic) networks. These networks operate in a state of “criticality” (marginal stability—Daniels et al., 2018) and thus their subversion may lead to a catastrophic failure of homeostasis, either locally (i.e., in the vicinity of the bite site) or systemically. On the other hand, organisms are buffered systems and the effects of interference with a single gene product are often minimal at the level of the integrated phenotype (Noble, 2011). The complexity of the venom cocktail, therefore, is in service of the overall function of the trait–venoms typically attack multiple regulatory networks simultaneously and may attack each network on multiple fronts. This is also highlighted by the complexity of clinical envenoming syndromes in human bite victims (Gutiérrez et al., 2017).

According to the World Health Organisation, an estimated 2 million people are envenomed following a snakebite every year and approximately 100,000 of these cases are fatal (The Lancet, 2017). The majority of these cases occur in developing countries in sub-Saharan Africa, South Asia and South East Asia (Gutiérrez et al., 2017; Longbottom et al., 2018). Despite Australia’s reputation for deadly snakes, snakebite envenoming is far less frequent on that continent. However, deaths do occur there every year because of snakebite and brown snakes (genus *Pseudonaja*) are the most “medically significant” snake taxa, being responsible for 41% of all deaths from snakebite envenoming between 2005 and 2015 (Johnston et al., 2017).

Patients who are envenomed by *Pseudonaja* spp. may exhibit a range of clinical symptoms, including venom-induced consumption coagulopathy (VICC), hypotension or cardiovascular collapse. Despite the presence of both pre- and post-synaptic neurotoxins in the venom, paralysis is only a rare consequence of brown snake envenoming, a fact known as the “brown snake paradox” (Barber et al., 2012). VICC is the most common severe symptom observed in cases of human envenoming by *Pseudonaja* spp., though hypotension and subsequent cardiovascular collapse are also potentially life-threatening (Allen et al., 2012). Unlike VICC, which is caused by the prothrombinase toxin pseutarin C interfering with the coagulation cascade (Rao and Kini, 2002), the mechanisms underlying the induction of hypotension and collapse following snakebite remain contentious, although pseutarin C has also been implicated in these pathologies (Chaisakul et al., 2015). Hypotensive effects of snake venoms have also been attributed to the release of dilator autacoids (Chaisakul et al., 2014), blockade of L-type voltage-gated Ca^2+^ channels (Yagami et al., 2013), presence of bradykinin-potentiating (Ferreira et al., 1970; Ianzer et al., 2004; Camargo et al., 2012) and natriuretic (Fry et al., 2005; St Pierre et al., 2005) peptides, or as a side effect of disseminated intravascular coagulation (Tibballs et al., 1992).

The venoms of *Pseudonaja* spp. are notably diverse in toxin content and highly variable–intra-as well as interspecific variations have been documented (Flight et al., 2006; Jackson et al., 2016; Reeks et al., 2016). Diversity exists both in terms of the variety of toxin classes present within a given venom, as well as within toxin classes, such as post-synaptically neurotoxic “3-finger toxins” (3FTx), which may exist in more than 40 variants within a single venom (Jackson et al., 2013). As well as the prothrombinase homolog pseutarin C (Rao and Kini, 2002), the venoms of *Pseudonaja* spp. contain phospholipases A_2_ (PLA_2_), including the presynaptic neurotoxin textilotoxin (Su et al., 1983); long chain and short chain 3FTx including the post-synaptic neurotoxins pseudonajatoxin a (Barnett et al., 1980) and b (Tyler et al., 1987); serine protease inhibitors including textilinin-1 and -2 (Masci et al., 2000); cysteine-rich secretory peptides; and vasoactive C-type natriuretic peptides (Judge et al., 2002; Birrell et al., 2006; Viala et al., 2015; Jackson et al., 2016; Reeks et al., 2016).

PLA_2_ toxins exhibit diverse activities, including neurotoxicity (Su et al., 1983; Lambeau et al., 1989); myotoxicity (Harris and MacDonell, 1981; Dixon and Harris, 1996); procoagulant (Judge et al., 2002); synergistic/auxillary interactions with other toxins (Bougis et al., 1987; Mukherjee, 2010; Mora-Obando et al., 2014; Laustsen, 2016); and others, including both enzymatic and non-enzymatic activities (Kini, 2003). Inhibition of PLA_2_ toxins, therefore, may have considerable therapeutic benefit for patients suffering from snakebite envenoming, including those envenomed by Australian brown snakes. Lewin et al. (2016) found that varespladib–a secretory phospholipase A_2_ (sPLA_2_) inhibitor–inhibited the phospholipase activities of 28 snake venoms. Further, rats treated with varespladib before or after envenoming had higher survival rates. Its pro-drug methyl-varespladib, when administered in conjunction with antivenom, also rescued mice administered otherwise lethal doses of *Oxyuranus scutellatus* venom (Lewin et al., 2018). These studies have revealed the potential of varespladib as an adjunct therapy, to be administered along with antivenom and/or additional small molecule therapeutics, for snakebite envenoming.

In the present study, we examined the cardiac, vascular, and sympathetic effects induced by five *Pseudonaja* spp. venoms in rat isolated atria and small mesenteric arteries. Where the brown snake venoms caused significant effects, the role of sPLA_2_s in mediating these responses was assessed by treatment with varespladib *in vitro*. Further, the role of nitric oxide, prostaglandins or CGRP in venom-induced vascular relaxation was investigated.

## 2 Materials and Methods

### 2.1 Materials

Venoms from five of the nine species comprising the genus *Pseudonaja* were examined in this study: *P. affinis* (dugite), *P. aspidorhyncha* (strap-snouted brown snake), *P. inframacula* (Peninsula brown snake), *P. textilis* (common or eastern brown snake) and *P. nuchalis* (northern brown snake). Venoms of the first four brown snake species were collected from South Australian specimens, whereas *P. nuchalis* venom was from a Northern Territory specimen. Lyophilised venoms were obtained from Venom Supplies Pty. Ltd. (Tanunda, South Australia) and diluted in phosphate-buffered saline (PBS; Oxoid Ltd., Hampshire, England) to a w/v ratio of approximately 10 mg/ml. Actual protein concentrations for each venom batch were determined by a Bradford protein assay, and subsequently all venom concentrations were expressed as µg/ml of protein concentration.

Drugs and suppliers were as follows: acetylcholine bromide (Sigma-Aldrich, St. Louis, MO, United States); benextramine tetrahydrochloride (Sigma); calcitonin gene-related peptide (human) 8–37 (CGRP_8–37_; Synpeptide Co. Ltd., Shanghai, China); 9,11-dideoxy-9α,11α-methanoepoxy-prosta-5Z,13E-dien-1-oic acid (U46619; Cayman Chemical, Ann Arbor, MI, United States); indomethacin (Sigma); (-)-isoprenaline (+)-bitartrate salt (Sigma); N_ω_-nitro-L-arginine methyl ester hydrochloride (L-NAME; Sigma); noradrenaline bitartrate (Sigma); prazosin hydrochloride (Sigma); tetrodotoxin (Sigma); and varespladib (LY315920; Tocris Bioscience, Bristol, United Kingdom). All drug dilutions were in Milli-Q grade water (15 MΩ.cm), purified using the Elix^®^ Essential Water Purification System (Merck Millipore, Darmstadt, Germany), except for varespladib and indomethacin, which were dissolved in 100% dimethyl sulfoxide (DMSO) or 0.01 M sodium bicarbonate, respectively.

### 2.2 Tissue Collection

Experiments were approved by the University of Melbourne Animal Ethics Committee and performed in accordance with the *Australian code for the care and use of animals for scientific purposes* (National Health and Medical Research Council, Canberra, Australia, 2013). Male Sprague-Dawley rats (250–350 g) were placed in a secure box and deeply anaesthetised by inhalation of isoflurane 5% (Baxter Healthcare Pty Ltd., New South Wales, Australia) in 95% O_2_ and killed by decapitation. Tissues were isolated, placed in a Silastic-coated petri dish and immersed in cold physiological salt solution (PSS) of the following composition (in mM): NaCl 119, KCl 4.69, MgSO_4_ 1.17, KH_2_PO_4_ 1.18, glucose 11 (5.5 for the myography preparations), NaHCO_3_ 25, EDTA 0.026 and CaCl_2_ 2.5; with pH 7.4.

### 2.3 Left and Right Atria

The left atrium and the spontaneously beating right atrium were separated from each other and pinned using two stainless steel hooks. The atria were then mounted in warm (37°C) PSS-filled 15 ml organ baths aerated with carbogen (95% O_2_, 5% CO_2_) for the duration of the experiment. Each atrium was attached to a Grass FT03C force transducer (Grass Instrument Co., Quincy, MA, United States) connected to a 6-channel amplifier (Octal Bridge Amp, ADInstruments, Sydney, Australia). Data were acquired by LabChart Acquisition Software (v7.0; ADInstruments). The atria were then stretched to 0.5 g and equilibrated for 10 min before another re-stretch to 0.5 g. The left atrium was electrically stimulated at 1 Hz, 0.25 ms at 150% threshold voltage via two punctate electrodes connected to a Grass S88 stimulator, while the right atrium was spontaneously beating. Tissues were allowed a 30 min equilibration period before their viability was tested with the β-adrenoceptor agonist isoprenaline (right atrium: 0.01 µM, left atrium: 0.1 µM). Tissues were washed with warm PSS and treated with one of *P. textilis*, *P. affinis*, *P. inframacula* or *P. nuchalis* venom in half-log_10_ cumulative increments (venom protein concentration 0.1–30 µg/ml). *P. aspidorhyncha* venom was not tested in the atrial bioassays.

### 2.4 Mesenteric Arteries

Second or third order arteries (i.d., 200–400 µm; 2 mm length segments) were dissected from the mesenteric vasculature and mounted in wire myograph chambers (Model 610M, Danish Myo Technology, Aarhus, Denmark). Vessels were submerged in 6 ml of PSS at 37°C and aerated with carbogen (95% O_2_, 5% CO_2_) for the duration of the experiments. To ensure that all mesenteric arteries were subjected to the same experimental conditions and optimal force development, each underwent a normalisation process by passive stretch as described by Angus and Wright (2000). Thirty min after the normalisation process, the PSS was replaced with 6 ml of potassium physiological salt solution (KPSS, with an equimolar substitution of KCl for NaCl^−^K^+^ 124 mM) to obtain each vessel’s depolarising maximum contraction response. These KPSS maximal responses were used as a reference point. To check that the endothelium layer remained intact, arteries were contracted with noradrenaline (3 µM) and tested for relaxation with acetylcholine (1 µM); only vessels with more than 50% relaxation were used in this study.

#### 2.4.1 Vascular Relaxant Effects of Venoms

To examine whether *Pseudonaja* spp. venoms induce vasorelaxation, arteries were pre-contracted with the thromboxane A_2_ mimetic U46619 (100 nM) to approximately 80% of their KPSS-induced maximal contraction and a single concentration of a venom (30 µg/ml) was added. In sPLA_2_ inhibition studies, arteries were incubated with 1 µM of varespladib (Lewin et al., 2016) for 30 min before exposure to U46619. At the plateau contraction to U46619, a venom (30 µg/ml) was added. The protocol of using only a single concentration of each venom was chosen due to rapid desensitization in vascular relaxation responses observed with various Australian elapid snake venoms (Chaisakul et al., 2012; Chaisakul et al., 2013; Chaisakul et al., 2014). In a separate set of experiments, *P. textilis* venom (30 µg/ml) was tested after pretreatment (30 min; literature supporting each chosen antagonist concentration shown) with one of 1) the CGRP antagonist CGRP_8–37_ (3 µM) (Wisskirchen et al., 1998); 2) the cyclooxygenase inhibitor indomethacin (3 µM) (Kassab et al., 2017); or 3) the nitric oxide synthase inhibitor L-NAME (100 µM) (Angus et al., 2017).

#### 2.4.2 Effects of Venoms on Sympathetic Nerve-Mediated Vascular Contractions

**FIGURE 1:**
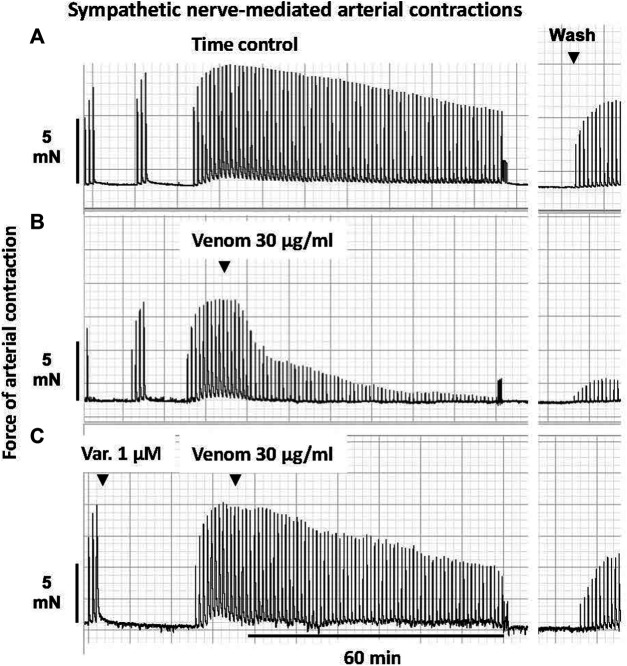
Representative computer traces of the sympatholytic effects of *Pseudonaja affinis* venom in the absence or presence of the sPLA_2_ inhibitor varespladib in rat isolated electrically stimulated small mesenteric arteries. **(A)** Time control–no venom or inhibitor treatment. **(B)** The addition of *P. affinis* venom (30 µg/ml) markedly inhibited nerve-mediated contractions **(C)** Pre-incubation with varespladib 1 µM (Var.) attenuated the sympatholytic effects of *P. affinis* venom. Wash, after 30 min of repeated washout of the bath solution, nerve stimulation responses were retested; very little recovery of contractile responses was observed after venom treatment alone **(B)**.

Arteries in myograph chambers were stimulated via platinum electrodes connected to a low-output-resistance stimulator (Grass S88). After the viability procedure described above, a test stimulation (3 s train duration every min, 25 Hz, 0.25 ms, 30 V dial setting) was conducted (Figure 1). Tissues were then incubated with 100 nM of prazosin for 5 min (to protect α_1_-adrenoceptors), followed by 30 µM of benextramine (an irreversible α_1_- and α_2_-adrenoceptor antagonist) for another 5 min to enhance the sympathetic-mediated responses of small resistance arteries via the blockade of presynaptic α_2_-adrenoceptors (Angus et al., 1988). Arteries were then washed with warm PSS every 5 min for 30 min, then exposed to 10 µM noradrenaline to ascertain that α_1_-adrenoceptor-mediated contractions were present; the noradrenaline was then washed out. Only arteries with a nerve stimulation contractile response of at least 50% KPSS were used. After 10 min, arteries were continuously stimulated until plateau, then a brown snake venom (30 µg/ml) was added, and nerve stimulation continued for 60 min.

In sPLA_2_ inhibition studies, arteries were incubated with varespladib (1 µM) for 30 min and then continuously stimulated. Following a plateau in the contractile response, 30 µg/ml of a brown snake venom was added. Nerve stimulation responses were recorded for a further 60 min. An example of this protocol is shown in Figure 1. Tetrodotoxin (0.3 µM) was used to ascertain that vascular smooth muscle was not directly stimulated.

### 2.5 Data and Statistical Analyses

All data are presented as the mean ± standard error of the mean (SEM) of *n* experiments (each tissue from a separate rat). Responses in left or right atria are expressed as the change (Δ) from resting baseline in contractile force (g) or in atrial rate (beats/min), respectively, within each tissue. Contractile responses in arteries are expressed as the percentage of the maximum reference contraction to KPSS (%KPSS), or as a percentage of the U46619-induced precontractile baseline tone, within each artery. For sympathetic nerve stimulation experiments, contractions in arteries are expressed as a percentage of the initial twitch height within each tissue.

Data were plotted and analysed using Prism 8 (GraphPad Software, La Jolla, CA, United States). For comparison of values between >2 treatment groups, one-way ANOVA with Dunnett’s or Tukey’s *post hoc* test, as appropriate, for multiple pairwise comparisons was performed. Responses in two groups were compared using a two-tailed Student’s unpaired *t* test, while paired responses within a group were compared with a Student’s paired *t* test. Within treatment group, atrial responses to increasing concentrations of venom were compared using repeated measures one-way ANOVA with Greenhouse-Geisser correction for correlation. In arteries, the effects of sympathetic nerve stimulation over time and between venom/vehicle treatments were compared using repeated measures two-way ANOVA with Greenhouse-Geisser correction for correlation and Dunnett’s *post hoc* test for multiple comparisons. Statistical significance was taken as *p* < 0.05.

## 3 Results

### 3.1 Cardiac Effects of Brown Snake Venoms

**FIGURE 2:**
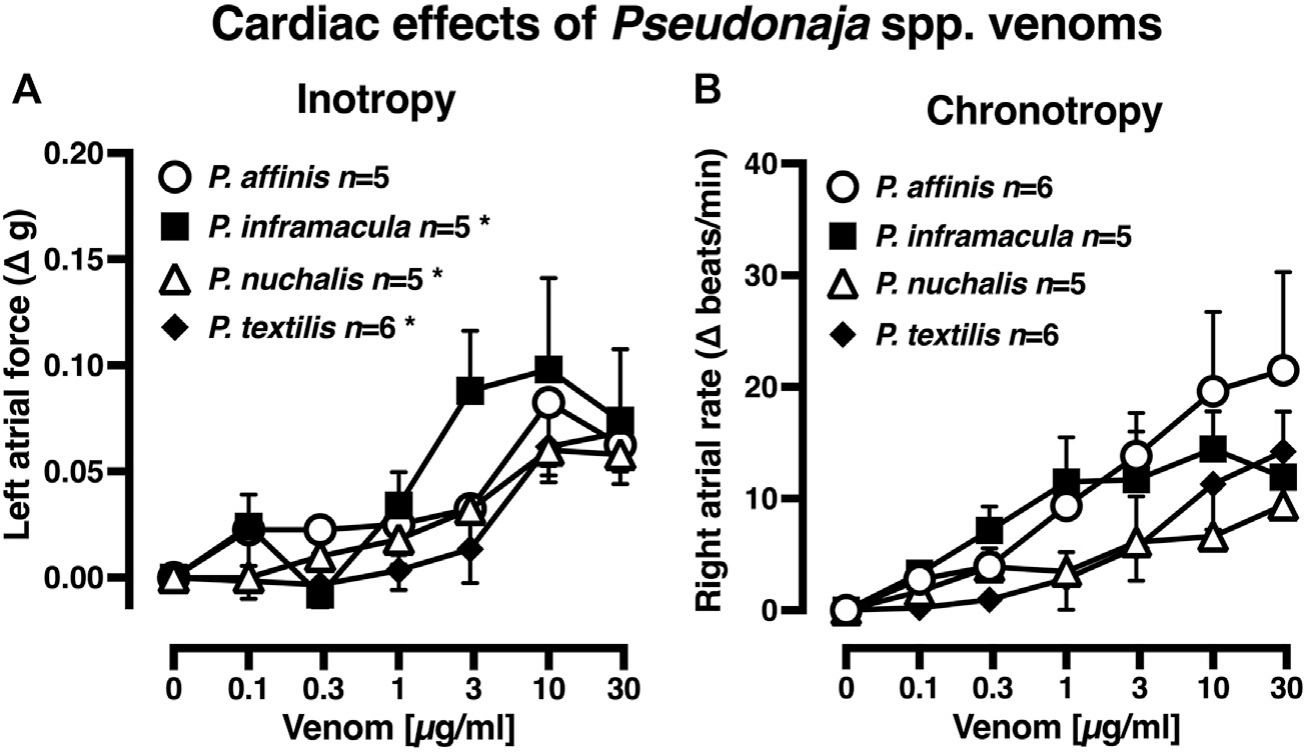
Effects of *Pseudonaja* spp. venoms (0.1–30 µg/ml) on **(A)** force of contraction of rat isolated left atria (responses are shown as change (Δ) in force (g) from baseline); and **(B)** rate of spontaneously beating isolated right atria (responses are Δ in atrial rate (beats/min) from baseline). Data are mean ± 1 SEM from *n* tissues from separate rats. *p < 0.05, repeated measures one-way ANOVA within treatment group over venom concentration range.

There were no significant differences between the baseline levels of contractility (left atria) or spontaneous atrial rate (right atria) between tissues allocated to the four venom treatment groups (*p >* 0.05, one-way ANOVA with Tukey’s *post hoc* test; data not shown). In rat isolated left atria, treatment with *P. inframacula, P. nuchalis* or *P. textilis* venoms elicited small increases in contractility at 10–30 µg/ml (+0.06–0.1 g, *n =* 5, 6; *p* < 0.05, repeated measures ANOVA; Figure 2A). To put this in context, these small positive inotropic responses were <10% of the pooled left atrial maximum contractile response to isoprenaline 0.1 µM of 1.02 ± 0.07 g (*n* = 21).

In isolated right atria, none of the four *Pseudonaja* venoms (0.1–30 µg/ml) had any significant effect on rate (Figure 2B; *p* > 0.05, repeated measures ANOVA). The pooled right atrial maximum chronotropic response to isoprenaline 0.01 µM was an increase from baseline of 139.0 ± 11.4 beats/min (*n* = 22). The venom of *P. aspidorhyncha* was not tested in atria due to the lack of physiologically significant effects of the other four *Pseudonaja* spp. venoms.

### 3.2 Vascular Effects of Brown Snake Venoms

The values of artery internal diameter (µm), contraction to depolarising PSS (KPSS; mN) and initial contraction (mN) when pre-contracted with U46619 or electrically stimulation were all consistent in arteries across the treatment groups (*p >* 0.05, one-way ANOVA; data not shown).

#### 3.2.1 Vasorelaxant Effects of Brown Snake Venoms

**FIGURE 3:**
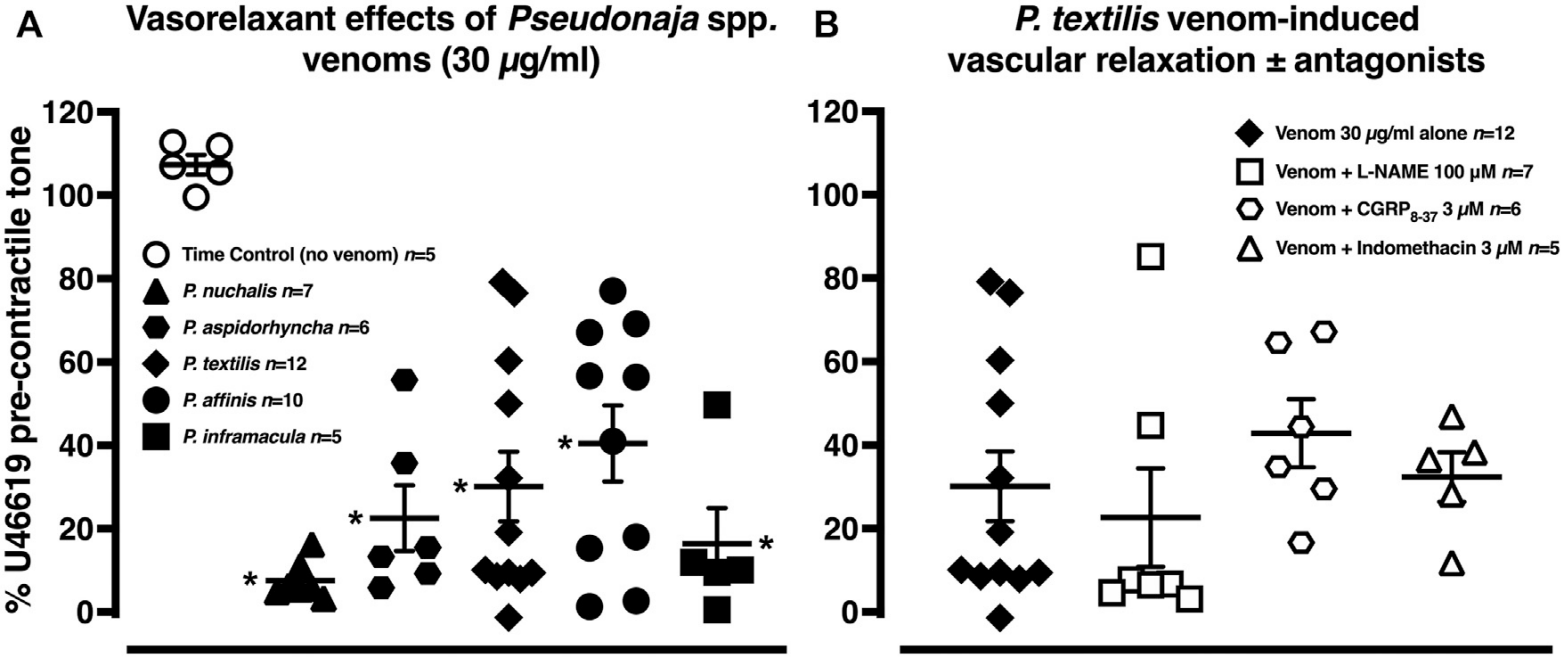
**(A)** Vascular relaxant effects of *Pseudonaja* spp. venoms (each 30 µg/ml) in rat isolated mesenteric arteries pre-contracted with U46619 (0.1 µM). **(B)** Vasorelaxation effects of *Pseudonaja textilis* venom (30 µg/ml) in the absence or presence of antagonists of nitric oxide synthase (L-NAME 100 µM), calcitonin gene-related peptide (CGRP_8–37_ 3 µM) or cyclooxygenase (indomethacin 3 µM). Data are values of individual experiments expressed as a % of the pre-contractile U46619 tone. Horizontal bars are mean and vertical bars ± 1 SEM of *n* arteries from separate rats. *p < 0.05 vs time control (no venom), one-way ANOVA with Dunnett’s *post hoc* test.

In the time control (no venom) group, the pre-contractile U46619 tone remained stable (Figure 3A; *n* = 5, *p >* 0.05, two-tailed paired Student’s *t* test). The five brown snake venoms (30 µg/ml) all elicited marked vasorelaxation of −60–−92% of pre-contractile tone (Figure 3A, *n =* 5–12; *p <* 0.0001 compared with time control group, one-way ANOVA with Dunnett’s *post hoc* test).

The vasorelaxant effects of *P. textilis* venom (30 µg/ml) were explored further in separate experiments. There was no attenuation of these effects with antagonism of calcitonin gene-related peptide (CGRP) receptor, cyclooxygenase or nitric oxide synthase (Figure 3B, *n =* 5–7; *p >* 0.05, one-way ANOVA with Dunnett’s *post hoc* test), suggesting little role for CGRP, dilator prostaglandins or nitric oxide in the venom-induced vasorelaxation.

**FIGURE 4:**
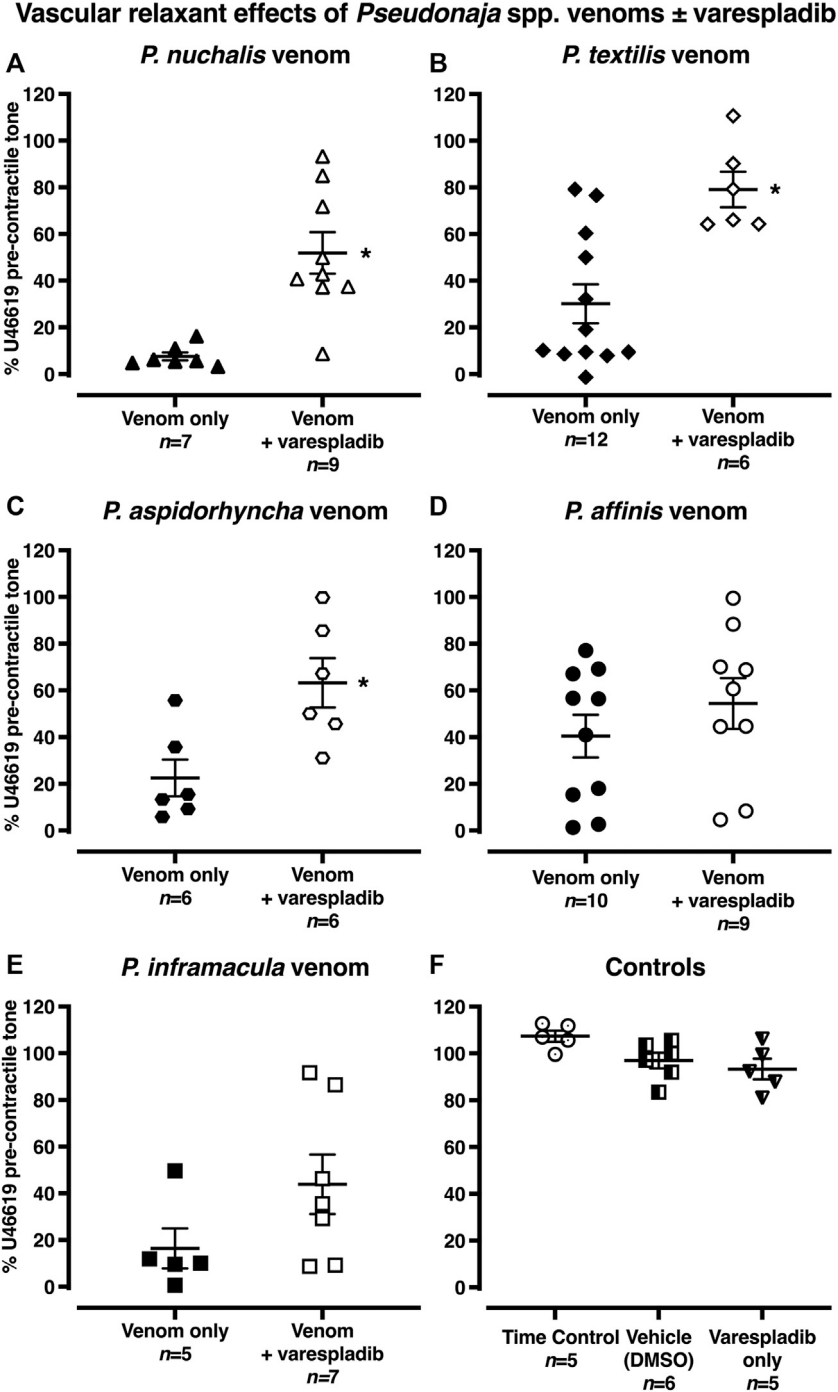
**(A–E)** Vascular relaxant effects of *Pseudonaja* spp. venoms (30 µg/ml) in the absence or presence of varespladib (1 µM) in rat isolated small mesenteric arteries. Responses are expressed as % of U46619 pre-contractile tone and individual values are shown. **(F)** Controls: time control-arteries did not receive any treatment; vehicle (DMSO)-arteries were only treated with DMSO (0.1% in bath); or varespladib only-arteries were treated only with varespladib (1 µM in 0.1% DMSO in bath). Horizontal bars are mean and vertical bars ± 1 SEM of *n* arteries from separate rats. *p < 0.05 vs the respective venom only group, Student’s unpaired *t* test.

After pre-treatment with the sPLA_2_ inhibitor varespladib (1 µM), vascular relaxant effects of *P. nuchalis*, *P. aspidorhyncha* and *P. textilis* venoms were significantly attenuated (Figure 4). In the presence of varespladib, the vasorelaxation effects of *P. nuchalis* were −48 ± 9% of pre-contractile tone (*n =* 9), significantly less than the −92 ± 2% relaxation seen in the venom only group (*n =* 7, Figure 4A; *p =* 0.0007, unpaired Student’s *t* test). Varespladib also significantly attenuated the vasorelaxant effects of *P. textilis* venom (−21 ± 8%, *n =* 6, vs. −70 ± 8% venom alone, *n =* 12, Figure 4B; *p =* 0.0016) and *P. aspidorhyncha* venom (−37 ± 11% vs −78 ± 8% venom alone, *n =* 6 each, Figure 4C; *p =* 0.012, unpaired Student’s *t* test). Varespladib did not affect relaxation responses to *P. affinis* or *P. inframacula* venoms (*p >* 0.05; Figures 4D,E). Treatment with vehicle (0.1% DMSO, *n =* 6) or varespladib alone (*n =* 5) did not affect contractile responses in rat isolated mesenteric arteries (*p >* 0.05, compared with time control group, one-way ANOVA with Dunnett’s *post hoc* test; Figure 4F).

#### 3.2.2 Sympatholytic Effects of *Pseudonaja* spp. Venoms in Mesenteric Arteries

**FIGURE 5:**
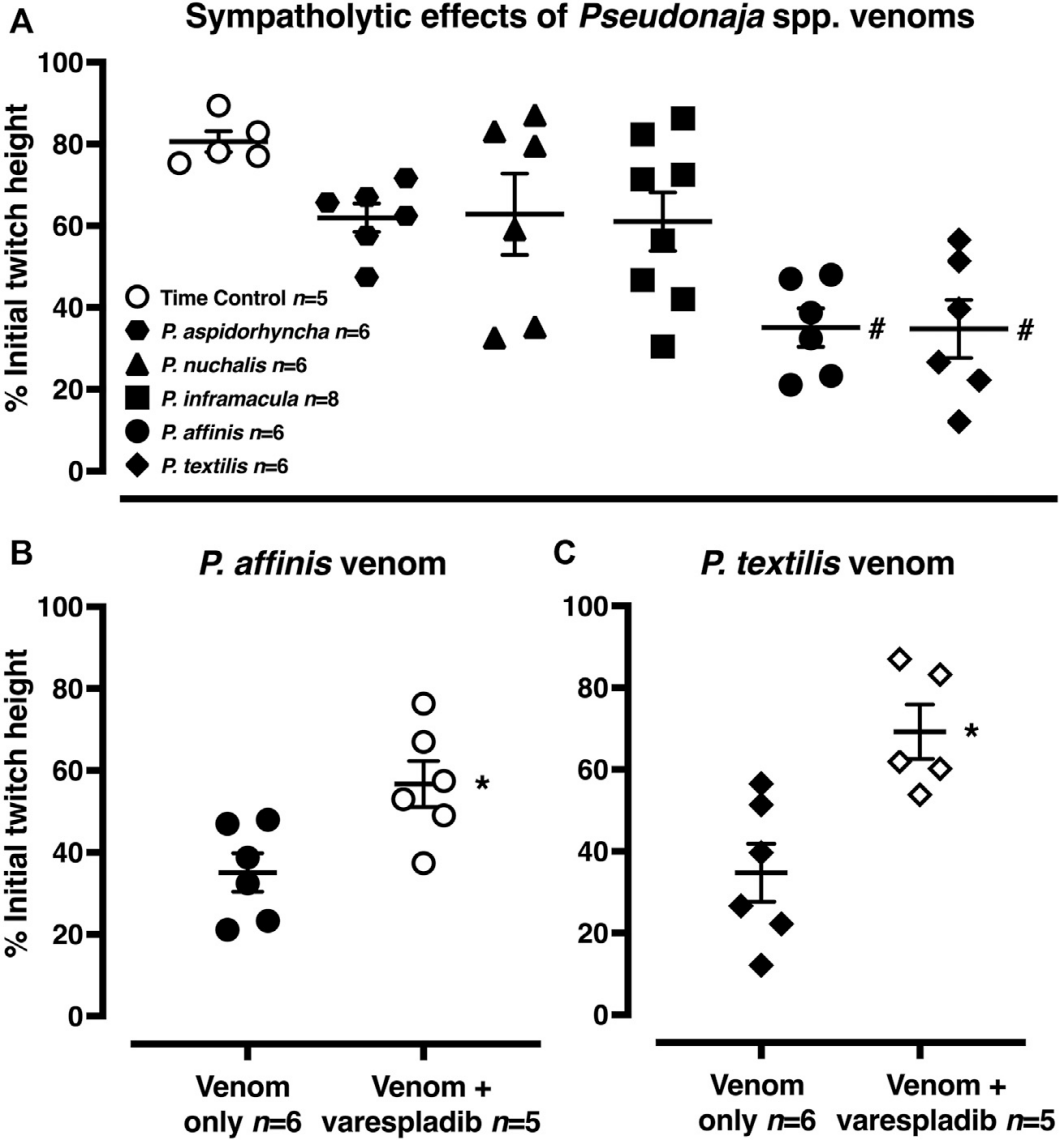
**(A)** Sympatholytic effects of five *Pseudonaja* spp. venoms (30 µg/ml each) 20 min after administration in rat isolated electrically stimulated small mesenteric arteries. **(B)** The sympatholytic effects of *P. affinis* and **(C)** *P. textilis* venoms (30 µg/ml) in the absence (venom only) or presence of varespladib (1 µM). Responses are expressed as % of the initial electrically induced contractile twitch height (at 0 min) and individual values are shown. Horizontal bars represent the mean and vertical bars ± 1 SEM of *n* arteries from separate rats. ^#^ *p <* 0.05 vs time control, one-way ANOVA with Dunnett’s *post hoc* test. *p < 0.05 vs. respective venom only groups, unpaired Student’s *t* test.

In the time control group, 20 min after the electrically stimulated contraction had reached a plateau, there was a decrease of −19 ± 3% of the initial twitch height (i.e., to 81 ± 3% of initial contraction, *n =* 5, Figures 1, 5A; *p <* 0.0001, repeated measures two-way ANOVA with Dunnett’s *post hoc* test). Of the five *Pseudonaja* spp. venoms (each 30 µg/ml), only *P. affinis* and *P. textilis* caused significant inhibition of nerve-mediated contractions (Figure 5A). *P. affinis* venom induced sympatholytic effects of −65 ± 5% of initial twitch height (*n =* 6, *p <* 0.0001 vs time control). Likewise, arteries treated with *P. textilis* venom were inhibited by −65 ± 7% (*n =* 6, *p <* 0.0001 vs time control). The administration of the other three venoms caused similar decreases in electrically induced contractions of −38 ± 3% (*P. aspidorhyncha*, *n =* 6), −37 ± 9% (*P. nuchalis*, *n =* 6) and −39 ± 7% (*P. inframacula*, *n =* 8), however these values were not significantly different to the time control group (*p =* 0.24, *p =* 0.28 and *p =* 0.15, respectively; one-way ANOVA with Dunnett’s *post hoc* test; Figure 5A).

The effects of the sPLA_2_ inhibitor varespladib were tested against the significant sympatholytic effects of both *P. affinis* (Figures 1, 5B) and *P. textilis* (Figure 5C) venoms. Sympathetic nerve-mediated contractions in arteries that were exposed to varespladib and *P. affinis* venom were significantly greater than in the venom only group (−43 ± 6% vs. −65 ± 5%, respectively, *n =* 6 each, Figure 5B; *p =* 0.014). Varespladib treatment also caused a significant attenuation of the sympatholytic effects of *P. textilis* venom, as the contractile responses of arteries incubated with the sPLA_2_ inhibitor increased to −31 ± 7% of the initial twitch height (*n =* 5), compared to those treated with venom only (−65 ± 7%, *n =* 6, Figure 5C; *p =* 0.007, unpaired Student’s *t* test).

## 4 Discussion

The results of the present study highlight the variability of brown snake venoms, both in terms of their baseline toxicity and their relative inhibition with the sPLA_2_ inhibitor varespladib. They also contribute additional evidence in support of the potential utilisation of varespladib as an adjunct therapy for snakebite envenoming. Perhaps their most important contribution is to the investigation of the cardiovascular effects of these venoms, which have largely been neglected, despite cardiac arrest being the most common cause of death following snakebite envenoming in Australia (Allen et al., 2012; Johnston et al., 2017). Few studies have concentrated on deconvoluting the causes of cardiovascular collapse following brown snake envenoming, with the notable exceptions of *in vitro* (Chaisakul et al., 2015) and *in vivo* (Chaisakul et al., 2013) studies of the effects of *P. textilis* venom. Interestingly, in the present study we found little (*P. inframacula, P. nuchalis* and *P. textilis*) to no (*P. affinis*) positive inotropic effects, and no effects on chronotropy, induced by the four brown snake venoms tested. These results corroborate those of the *in vivo* study of *P. textilis* venom (Chaisakul et al., 2013), as well as one conducted by the same team with isolated sPLA_2_s from *Oxyuranus scutellatus* (taipan) venom (Chaisakul et al., 2014). Collectively, these results suggest that venom-induced cardiovascular collapse following envenoming by Australian snakes does not result from direct cardiac toxicity but is more likely to be mediated by effects in the vasculature.

Chaisakul et al. (2013) examined the pharmacology of whole *P. textilis* venom and found that it caused vasorelaxation. Our results agree with this finding for *P. textilis* and demonstrate that vasorelaxant activity is a marked effect of the venoms of four additional species of *Pseudonaja* (i.e., *P. affinis, aspidorhyncha, inframacula* and *nuchalis*), suggesting it is a widespread property of brown snake venoms. Inhibition with varespladib significantly attenuated the vasorelaxant effects of three of the venoms (*P. nuchalis, textilis* and *aspidorhyncha*), thus our results indicate that sPLA_2_s of these species may functionally antagonise vasocontraction (vascular tone) in mammalian arteries. However, varespladib (1 µM) pretreatment did not significantly inhibit vasorelaxant effects of *P. affinis* or *P. inframacula* venoms, suggesting that these effects may be mediated by a combination of toxins which are present in differing ratios in each of the venoms. One of the limitations of our study was the protocol of using only a single venom concentration, rather than the construction of a concentration-response curve for each *Pseudonaja* venom. However, it has been previously reported for both *P. textilis* and *Oxyuranus scutellatus* (Papuan taipan) venoms that rapid desensitization occurs with repeated exposure of isolated arteries *in vitro* (Chaisakul et al., 2012; Chaisakul et al., 2013; Chaisakul et al., 2014). The use of only a single venom concentration per tissue was therefore considered the most rigorous pharmacological approach.

Although our study did not examine the cardiovascular effects of isolated toxin fractions, the sPLA_2_-mediated vasorelaxant activity *of Oxyuranus scutellatus* (coastal taipan) venom has previously been investigated in a similar bioassay (Chaisakul et al., 2014). *Oxyuranus* is the sister genus to *Pseudonaja* and the venoms of both genera contain closely related toxins, a number of which are unique to the *Oxyuranus/Pseudonaja* clade (Jackson et al., 2016).

A fraction containing pseutarin C, the procoagulant toxin in *P. textilis* venom, was also found to induce a significant fall in mean arterial pressure without altering cardiac function (Chaisakul et al., 2015). *In vitro* experiments from the same study demonstrated that pseutarin C did not possess any vasorelaxant activity, further reinforcing the role of sPLA_2_s in the induction of vasorelaxation in mesenteric arteries. Taken together, these results indicate that multiple components–in this case, a prothrombin activator and sPLA_2_s (perhaps in addition to C-type natriuretic peptides also present in these venoms; St Pierre et al., 2006)–may contribute to the overall cardiovascular symptoms of snake envenomation. Notably, synergistic (potentiating) effects between PLA_2_s and other venom components have been described previously in a number of snake venoms, including those of the cobras *Naja mossambica* and *Hemachatus haemachatus* (Louw and Visser, 1978), the pit viper *Bothrops asper* (Mora-Obando et al., 2014), and the taipan *O. scutellatus* (Herrera et al., 2016).

Another popular hypothesis about snake venom-induced vasorelaxation invokes the release of dilator autacoids, particularly histamine (Feldberg and Kellaway, 1937; Teixeira et al., 2003) and bradykinin (Ferreira et al., 1970; Xu et al., 2015), as the proximal cause. Although this has largely been investigated using the venoms of viperid snakes (family Viperidae), a study using crude *O. scutellatus* venom showed that pre-treating rats with the cyclooxygenase inhibitor indomethacin, but not atropine (muscarinic receptor antagonist), mepyramine (histamine H_1_ receptor antagonist) or the nitric oxide synthase inhibitor L-NAME, could prevent vasodilation and sudden collapse (Chaisakul et al., 2012). PLA_2_ enzymes cause the release of lysophospholipids and fatty acids via hydrolysing glycerophospholipids (Kini, 2003). Chaisakul et al. (2014) showed that sPLA_2_-mediated hypotensive effects of *O. scutellatus* venom were inhibited in rats administered with indomethacin prior to venom administration, concluding significant release of dilator autacoids. An earlier study of the PLA_2_ fraction from *V. russelli* venom showed that PLA_2_ relaxed rat isolated aortic rings in an endothelium-independent manner, ruling out a role for the release of endothelial autacoids such as histamine and bradykinin (Huang and Lee, 1985). They found that inhibition of soluble guanylate cyclase with the early antagonist methylene blue partly attenuated the PLA_2_-induced aortic relaxation, suggesting a role for cyclic GMP in this response. In rat isolated mesenteric arteries, the relaxation caused by Papuan taipan venom was shown by Chaisakul et al. (2012) to involve, in part, protein kinase A and large-conductance Ca^2+^-activated K^+^ channels. In our study, pretreatment with indomethacin, L-NAME or the CGRP receptor antagonist CGRP_8-37_ did not abrogate the vasorelaxant effects of whole *P. textilis* venom in isolated mesenteric arteries, suggesting that the relaxation was not due to local release of dilator prostanoids or nitric oxide, or sensory nerve-released CGRP; the possible involvement of cyclic GMP formation, protein kinase A or large-conductance Ca^2+^-activated K^+^ channels was not tested and warrant further experimentation.

Although studies investigating the structure and activity of the main components of *P. textilis* venom–particularly pseutarin C and the presynaptically neurotoxic sPLA_2_ textilotoxin–have been conducted for more than 50 years, the remaining members of the genus *Pseudonaja* have received far less attention. PLA_2_s appear to be an almost ubiquitous component of Australian elapid snake venoms (Jackson et al., 2016) and have been detected in every *Pseudonaja* venom system investigated to-date (see e.g., Jackson et al., 2013; Jackson et al., 2016; Reeks et al., 2016). PLA_2_s have been identified in *P. nuchalis* and *P. aspidorhyncha* venoms as “homologues of textilotoxin PLA_2_ subunits” (Reeks et al., 2016); and in *P. affinis* venom as a “toxic PLA_2_ variant” (Judge et al., 2002). The latter study assayed the activity of purified PLA_2_s from *P. affinis* venom and found they induced contraction in rat tracheae–an activity like the venoms of *O. microlepidotus* and *O. scutellatus*, which was hypothesised by the authors to result from the downstream release of leukotrienes.

In the present study, only *P. affinis* and *P. textilis* venoms markedly inhibited sympathetic nerve-mediated vascular contractions of isolated mesenteric arteries. These effects were significantly inhibited by pretreatment with varespladib, indicating that PLA_2_ toxins play an important role in inducing this sympatholysis. As no studies have been performed which assess the sympatholytic effects of isolated textilotoxin homologues, their involvement in the inhibition of sympathetic transmission is unknown. Furthermore, neither *P. nuchalis* nor *P. aspidorhyncha* venoms, which contain textilotoxin isoforms (Reeks et al., 2016), induced significant sympatholytic effects in our assays. These data raise several questions: whether only certain isoforms of textilotoxin can cause these effects in rat mesenteric arteries; if particular concentrations must be achieved (i.e., whether the absence of the effect for some venoms is a consequence of the relative abundance of this toxin type); if synergy with post-synaptic neurotoxins (i.e., 3FTx) is required; or indeed if this presynaptic neurotoxin is involved at all. As varespladib significantly attenuated the sympatholytic effects of *P. affinis* and *P. textilis* venoms, it is reasonable to infer that some forms of sPLA_2_ act at synaptic terminals in vascular smooth muscle. Alternatively, they may act post-synaptically at the α_1_-adrenoceptor, which would explain the lack of effects on sympathetic nerve stimulation-induced inotropy (mediated by β_1_-adrenoceptors) in rat left atria–in contrast to the effects in blood vessels–in this study. Whether sPLA_2_s affect the function of α_1_-adrenoceptors in vascular smooth muscle is not known, however, PLA_2_ has been shown to desensitize α_2_-adrenoceptors in platelets (Rao and White, 1989), and in isolated membrane preparations (Cohen et al., 1985). This latter effect on α_2_-adrenoceptors is not relevant to the current study as these receptors were irreversibly blocked in the mesenteric arteries used in the sympathetic nerve stimulation experiments. The precise reason for the differential effects in atria and arteries is therefore impossible to ascertain based on our data but represents an interesting question for further investigation.

Previous studies indicate that presynaptic neurotoxins in snake venoms can deplete acetylcholine release from motor neurons in skeletal muscle via the destruction of synaptic terminals (Chang et al., 1973; Su and Chang, 1984; Cavalcante et al., 2017) and vesicular membranes (Montecucco et al., 2009). To complicate the matter further, whilst a proteomic study of *P. affinis* venom did not detect any textilotoxin homologues (Judge et al., 2002), multiple studies (Judge et al., 2002; Jackson et al., 2016) have detected an abundance of proteins in the 12–14 kDa range–the expected size of venom sPLA_2_s following decomplexation–in *P. affinis* venom. Thus, it is possible that the sympatholytic effects induced by this venom are caused by unknown sPLA_2_s. More detailed investigation into the composition of *Pseudonaja* venoms is therefore warranted to ascertain which PLA_2_ toxins are responsible for the sympatholytic effects observed in the present study.

## 5 Conclusion

Whilst all five brown snake venoms examined in the present study induced vasorelaxant effects, only two venoms–*P. textilis* and *P. affinis*–induced marked sympatholytic effects. The activity of the venoms (and their relative inhibition with varespladib) thus varied amongst species at the level of gross activity, as well as more subtly in terms of the potency of each activity. These findings corroborate earlier studies (e.g., Flight et al., 2006; Jackson et al., 2016; Reeks et al., 2016) that have uncovered considerable variability (at both inter- and intraspecific levels) in the composition and activity of brown snake venoms. Thus, our data contribute to the ongoing investigation of these biologically fascinating and medically significant venoms. However, it would be premature at this stage to conjecture about the evolutionary origins (i.e., the function *sensu stricto*), or possible clinical relevance of the variability we have reported here. Based on their inhibition with the sPLA_2_ inhibitor varespladib, we infer that PLA_2_ toxins in *P. aspidorhyncha*, *P. nuchalis* and *P. textilis* venoms are involved in brown snake venom-induced vasorelaxation and the sympatholytic effects of *P. affinis* and *P. textilis* venoms. Observed vasodilatory effects were likely endothelium-independent, although determination of the exact mechanism of action was outside the scope of this study. Given that little to no direct cardiac effects were observed in our assays, the cause of cardiovascular collapse following brown snake envenoming is likely due to a fall in mean arterial pressure unrelated to cardiac function. Further proteomic and pharmacological studies remain necessary to resolve the mechanistic details of this dangerous venom-induced pathology. Finally, our results are promising regarding the potential role of varespladib as an initial (pre-referral) and/or adjunct (in combination with antivenom) therapeutic agent for brown snake envenoming.

## Data Availability

The raw data supporting the conclusion of this article will be made available by the authors, without undue reservation.
